# FAIM Opposes Aggregation of Mutant SOD1 That Typifies Some Forms of Familial Amyotrophic Lateral Sclerosis

**DOI:** 10.3389/fnins.2020.00110

**Published:** 2020-02-21

**Authors:** Hiroaki Kaku, Alexander V. Ludlow, Michael F. Gutknecht, Thomas L. Rothstein

**Affiliations:** ^1^Center for Immunobiology, Western Michigan University Homer Stryker M.D. School of Medicine, Kalamazoo, MI, United States; ^2^Department of Biomedical Sciences, Western Michigan University Homer Stryker M.D. School of Medicine, Kalamazoo, MI, United States

**Keywords:** Amyotrophic lateral sclerosis, SOD1, protein aggregation, protein disaggregation, fas apoptosis inhibitory molecule

## Abstract

Amyotrophic lateral sclerosis (ALS) is a progressive neurodegenerative illness that is unremittingly fatal and for which no effective treatment exists. All forms of ALS are characterized by protein aggregation. In familial forms of ALS, specific and heritable aggregation-prone proteins have been identified, such as mutant superoxide dismutase (SOD1). It has been suggested that a factor capable of preventing mutant SOD1 protein aggregation and/or disassembling mutant SOD1 protein aggregates would ameliorate SOD1-associated forms of familial ALS. Here we identify Fas Apoptosis Inhibitory Molecule (FAIM), a highly evolutionarily conserved 20 kDa protein, as an agent with this activity. We show FAIM counteracts intracellular accumulation of mutant SOD1 protein aggregates, which is increased in the absence of FAIM, as determined by pulse-shape analysis and filter trap assays. In a cell-free system, FAIM inhibits aggregation of mutant SOD1, and further disassembles and solubilizes established mutant SOD1 protein aggregates, as determined by thioflavin T (ThT), filter trap, and sedimentation assays. In sum, we report here a previously unknown activity of FAIM that opposes ALS disease-related protein aggregation and promotes proteostasis of an aggregation-prone ALS protein.

## Introduction

Amyotrophic lateral sclerosis (ALS, also known as Lou Gehrig’s Disease) is an unremitting neurodegenerative disease characterized by progressive weakness and death within 2–5 years (Yedavalli et al., 2018). ALS primarily affects upper and lower motor neurons eventually causing paralysis, but non-motor manifestations such as cognitive dysfunction and frontotemporal dementia can occur (Ferrari et al., 2011). Unfortunately, there is no cure nor useful treatment for the primary disease. Despite a full clinicopathological description of ALS by Charcot in 1874 (Mathis et al., 2019), causal factors remain elusive. The vast majority of cases are sporadic with no inciting insult or associated illness. The remaining 5–10% of cases are inherited and designated familial ALS (FALS) (Kurland and Mulder, 1955). Genetic studies have demonstrated the involvement of mutant *SOD1*, *TARDBP* (TDP-43), *C9orf72*, or *FUS* in about half the cases of FALS, with other genes, and unknown genes, pathogenically involved in the remaining cases (Mathis et al., 2019).

The heterogeneity of ALS (dominant sporadic vs. uncommon familial; dominant rapid progression vs. rare chronic course) has made elucidation of pathogenesis difficult. However, dysfunctional proteostasis is a common feature of all forms of ALS. ALS cases are always characterized by the presence of protein aggregates in the brain or spinal cord (Mizuno et al., 2006), and it is well known that protein aggregates manifest cellular toxicity (Wood et al., 2003). Familial ALS involving SOD1 is a case in point, wherein it has been shown that mutant SOD1 produces protein aggregation and that altered SOD1 enzymatic activity, low or high, is not responsible for pathogenesis (Bruijn et al., 1998), strongly suggesting it is aggregation of SOD1 itself (rather than SOD1 enzymatic activity) that produces/contributes to disease. For these reasons, much attention has focused on protein aggregation as an etiologic factor in human ALS disease, especially FALS. It has been suggested that discovery of an agent that could block or disrupt protein aggregation would ameliorate disease in ALS patients (Shorter, 2016). Unfortunately, there is a paucity of known proteins suitable for therapeutic consideration that can alter aggregation of any protein.

Fas Apoptosis Inhibitory Molecule (FAIM) is a unique, approximately 20 kDa protein that was cloned by differential display during a study of B cell apoptosis. We found that overexpression of FAIM produced resistance to Fas- (CD95-) mediated cell death and so it was termed, FAIM (Schneider et al., 1999). FAIM is widely expressed (Zhong et al., 2001). We and others have studied FAIM in a variety of situations, and protection against cell death has been confirmed in a number of cell types. Beyond B cells, FAIM opposes death receptor-induced apoptosis in HEK293T cells (Li et al., 2014), PC12 cells (Segura et al., 2007), and primary neurons (Segura et al., 2007). It also protects against stress-induced cell death in primary mesenchymal stem cells (Liu et al., 2017) and in several cell lines derived from multiple myeloma (Huo et al., 2013). Further, FAIM affects other cellular activities that involve protein degradation/loss (Sole et al., 2004; Kaku and Rothstein, 2009). Thus, we found FAIM constitutes an anti-apoptotic, pro-survival protein whose impact spans multiple cell types.

FAIM is highly conserved (Schneider et al., 1999), suggesting that it serves an essential role in cellular activity. The expression and evolution pattern of *faim* and the related *faim-Gm6432* gene suggested that FAIM might be involved more generally in cellular stress response pathways, rather than narrowly protecting against death receptor demise (Turner and Lysiak, 2008; Qiu et al., 2013; Durairajanayagam et al., 2015). This turned out to be correct and in the course of this work we found that loss of FAIM was associated with increased levels of stress-induced protein aggregation in FAIM-deficient cell lines and animals (Kaku and Rothstein, 2019). This raised the possibility that FAIM can act directly on dysfunctional proteins that aggregate. To address this possibility, we examined the effect of FAIM on protein aggregation, focusing on mutant SOD1-G93A that spontaneously forms protein aggregates and is implicated in familial ALS.

## Methods

### Cell Culture and Transfection

HeLa cells were obtained from the American Type Culture Collection (ATCC). HeLa cells were cultured in DMEM medium (Corning) containing 10% FCS, 10 mM HEPES, pH 7.2, 2 μM L-glutamine, and 0.1 mg/ml penicillin and streptomycin. Transfection was performed using Lipofectamine 3000, according to the manufacturer’s instructions (Invitrogen).

### Generation of FAIM Knockout Cells With CRISPR/Cas9

Guide RNA (gRNA) sequences for the human FAIM gene were designed using a CRISPR target design tool^1^ in order to target the exon after the start codon, as previously described (Ran et al., 2013). Annealed double strand DNAs were ligated into pSpCas9(BB)-2A-GFP (PX458) vector (Addgene) at the Bpi1 (Bbs1) restriction enzyme sites using the “Golden Gate” cloning strategy. The presence of insert was verified by sequencing.

Empty vector was used as a negative control. Transfection was performed using lipofection and a week after transfection, eGFP^+^ cells were sorted with an Influx instrument (Becton Dickinson), and seeded into 96 well plates. FAIM knockout clones were screened by limiting dilution and western blotting.

CRISPR-Cas9 oligonucleotides used for this work were as follows: human *FAIM* forward, CACCGACAGATCTCGTA GCTGTTTGGG; human *FAIM* reverse, AAACAAACAGC TACGAGATCTGTC.

### Plasmids

The following plasmids were obtained from Addgene (Stevens et al., 2010; Ran et al., 2013).

**Table:** 

pF146 pSOD1WTAcGFP1	#26407
pF150 pSOD1G93AAcGFP1	#26411
pSpCas9(BB)-2A-GFP (PX458)	#48138

### Pulse-Shape Analysis (PulSA)

WT and FAIM KO HeLa cells were transiently transfected with an eGFP-tagged native human SOD1 or aggregation-prone SOD1-G93A protein expression vector. Cells expressing eGFP-tagged SOD1-G93A protein were harvested at the indicated times and eGFP expression was analyzed on an LSR Fortessa (BD Pharmingen) flow cytometer for PulSA analysis to detect protein aggregates, as previously described (Ramdzan et al., 2012). Data was collected in pulse-area, height and width for each channel. At least 10,000 cells were analyzed.

### Filter Trap Assay (FTA)

WT and FAIM KO HeLa cells were transiently transfected with an eGFP-tagged native human SOD1 or aggregation-prone human SOD1-G93A protein expression vector, and fluorescently tagged cells were then harvested at 48 h. Cells were washed with PBS and then lysed in PBS containing 2% SDS, 1 mM MgCl_2_, protease inhibitor cocktail and 25 unit/ml Benzonase (Merck). Protein concentrations were quantified using 660 nm Protein Assay Reagent with Ionic Detergent Compatibility Reagent (IDCR) (Thermo Scientific). Equal amounts of protein extracts underwent vacuum filtration through a 0.2 μm pore size cellulose acetate membrane (GE Healthcare) for the detection of SOD1 aggregates using a 96 well format Dot-Blot apparatus (Bio-Rad). The membrane was washed twice with 0.1% SDS in PBS and western blotted using anti-GFP antibody (Cell Signaling Technology) to detect aggregated proteins. Cell free aggregates of SOD1-G93A were similarly applied to nitrocellulose membrane. In cell-free experiments, SOD1 protein was vacuum filtered as above, after which membranes were western blotted using anti-SOD1 antibody (Cell Signaling Technology) to detect aggregated proteins.

### Thioflavin T Fluorescence Assay

Fibril/aggregate formation of mutant SOD1-G93A (10 μM) was assessed in the presence or absence of FAIM (4 μM) by Thioflavin T (ThT, 20 μM) (Sigma-Aldrich) fluorescence using a Synergy Neo2 Multi-Mode Microplate Reader (Bio-Tek). Reader temperature was set at 37°C with continuous double orbital shaking at a frequency of 425 cpm at 3 mm between reads. Aggregation conditions required the presence of the reducing agent TCEP [tris(2-carboxyethyl)phosphine] (Sigma) at 20 mM and EDTA at 5 mM, in the presence of an extreme-temperature slippery PTFE Teflon^®^ beads (McMaster-Carr). ThT fluorescence intensity was measured using an excitation wavelength of 440 nm and an emission of 482 nm. Photomultiplier (PMT) gain was set at 75. Fluorescence measurements were made from the top of the plate, with the top being sealed with an adhesive plate sealer to prevent evaporation.

### Disaggregation Assays

SOD1-G93A (2 μM) pre-formed fibrils were incubated with 8 μM recombinant FAIM at 37°C for 2.5 h. Then, fibril status was determined by either ThT fluorescence, FTA, or by detecting proteins in the supernatants and in the pellets after sedimentation at 21,000 × g and western blotting.

### Western Blotting

Protein concentrations were determined using the 660 nm Protein Assay Reagent (Pierce). Protein samples in 1 × Laemmli buffer with 2-mercaptoethanol at 2.5% were boiled for 5 min. Equal amounts of protein for each condition were subjected to SDS-PAGE on an AnykD gradient gel (Bio-Rad) followed by immunoblotting with anti-SOD1 antibody (Cell Signaling) after wet transfer for 1 h to PVDF membrane (Bio-Rad) and blocking with non-fat dry milk.

### His-Tag Recombinant Protein Production

His-tag protein expression vectors were constructed using pTrcHis TA vector according to the manufacturer’s instructions. In brief, PCR amplified target genes were TA-cloned into the vector (Invitrogen) and inserted DNA was verified by sequencing (Genewiz). Proteins were expressed in TOP10 competent cells (Invitrogen) with IPTG at 1 mM and were purified using a Nuvia IMAC Nickel-charged column (Bio-Rad) on a NGC Quest chromatography system (Bio-Rad). Protein purity was verified using TGX Stain-Free gels (Bio-Rad) on ChemiDoc Touch Imaging System (Bio-Rad) and each protein was determined to be >90% pure.

After elution from a nickel-charged column, aggregation-prone SOD1 was generated by demetallization with EDTA under reducing conditions (Abdolvahabi et al., 2019). Purification was performed in the presence of guanidine HCl to induce dimer subunit disassociation, followed by three stage dialysis buffer exchange in the presence of EDTA at 5 mM to remove metal ions.

Oligonucleotides used in this work for cloning into pTrcHis TA vector were as follows: human *FAIM* forward, ATGACAGATCTCGTAGCTGTTTGG; human *FAIM* reverse, TTAACTTGCAATCTCTGGGATTTC; human *SOD1* forward, ATGGCGACGAAGGCCGTGTG; human *SOD1* reverse, TTA TTGGGCGATCCCAATTACAC. Sequence primers used in this work were as follows: for pX-458, TGGACTATCATATGC TTACCGTAACTTGAAAG; for pTrcHis TA, TATGGCTAG CATGACTGGT.

Work described herein was carried out with adherence to all institutional safety procedures.

### Generation of Pre-formed Protein Aggregates for Disaggregation Assays

SOD1-G93A fibrils were assembled in an Eppendorf ThemoMixer F1.5 with ThermoTop, as previously described (DeSantis et al., 2012), with minor modifications. SOD1-G93A (80 μM) fibrils were generated in assembly buffer (AB; 40 mM HEPES-KOH pH 7.4, 150 mM KCl, 20 mM MgCl_2_, and 1 mM dithiothreitol) plus 10% (v/v) glycerol for 16 h with agitation. Fibrils were recovered by centrifugation, washed and resuspended in assembly buffer for disaggregation assays. For all fibrils, generation was confirmed by ThT fluorescence. Fibrils were diluted to the requisite concentration for subsequent disaggregation reactions (Figure 3).

### Statistics

All quantitative data are expressed as mean ± SEM. ANOVA or, when appropriate, unpaired *t*-test was used for statistical determinations with GraphPad Prism 7 software. Values of *p* < 0.05 are considered statistically significant (^∗^*p* < 0.05, ^∗∗^*p* < 0.01 or ^∗∗∗^*p* < 0.001.

## Results

**FIGURE 1:**
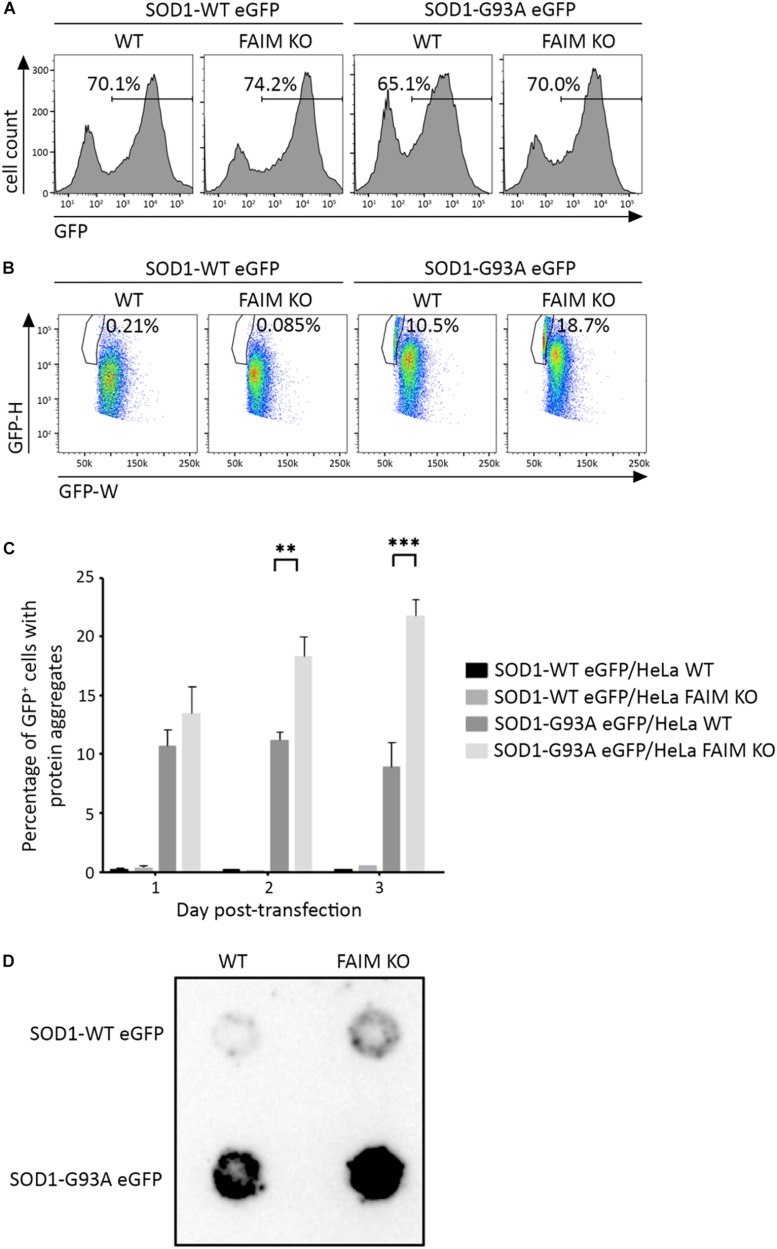
FAIM KO cells accumulate aggregation-prone mutant SOD1 protein. WT HeLa cells and FAIM KO HeLa cells were transiently transfected with expression vectors for native SOD1 and mutant SOD1-G93A that incorporate an eGFP tag. **(A,B)** 2 days later, eGFP^+^ cells **(A)** were selected and evaluated for pulse-width (GFP-W) vs. pulse-height (GFP-H) **(B)**. The gated area represents cells expressing aggregated proteins. Results representative of three independent experiments are shown. **(C)** WT and FAIM KO HeLa cells were transfected as in **(A)** and harvested at the indicated times. Percentages of cells expressing aggregated proteins out of total eGFP^+^ cells are shown. Data represent mean ± SEM from three independent experiments. **(D)** WT and FAIM KO HeLa cells were transfected as in **(A)**. After 2 days cells were lysed and equal amounts of total cell lysates were subjected to FTA and stained with anti-GFP. Similar results were obtained in at least three independent experiments.

To examine the activity of FAIM with respect to the disease-associated, aggregation-prone mutant protein, SOD1, we first deleted FAIM from HeLa cells by CRISPR/Cas9 excision (Kaku and Rothstein, 2019). We then transiently transfected FAIM-deficient HeLa cells and WT HeLa cells with eGFP-tagged mutant SOD1-G93A, which spontaneously forms aggregates. To directly assess the role FAIM plays in prevention of SOD1-G93A protein aggregation, we employed two assays. (1) We used pulse shape analysis (PulSA) by flow cytometry to determine the level of aggregated protein. In this assay, cells containing aggregated proteins have a narrower and higher pulse shape of eGFP fluorescence than those that do not express protein aggregates (Ramdzan et al., 2012). We found that regardless of transfection efficiency, the fraction of HeLa cells expressing aggregated mutant SOD1 was significantly higher in FAIM-deficient HeLa cells than in WT HeLa cells, especially at late stages after transfection (Figures 1A–C; Kaku and Rothstein, 2019). (2) We used filter trap assay (FTA) to evaluate the level of aggregated protein. In this assay, large aggregated proteins are not able to pass through a 0.2 μm pore-sized filter, remain on the filter, and are blotted with anti-GFP antibody (Myeku et al., 2011). We found that much more aggregated mutant SOD1 was filter trapped in FAIM-deficient HeLa cells than in WT HeLa cells (Figure 1D; Kaku and Rothstein, 2019). These results demonstrate the essential role of FAIM in blocking formation of mutant SOD1 aggregates.

**FIGURE 2:**
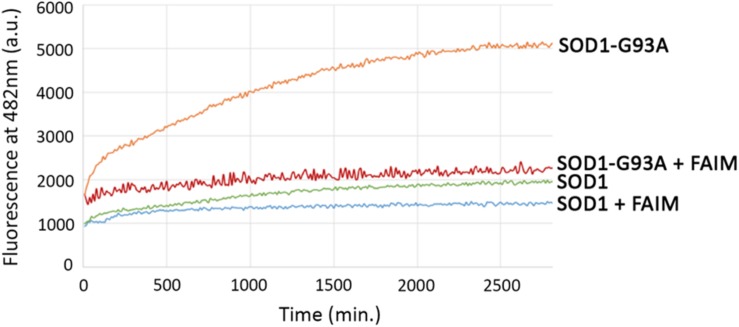
Recombinant FAIM prevents mutant SOD1-G93A aggregation in a cell-free system. Spontaneous aggregation of WT SOD1 and mutant SOD1-G93A (10 μM) *in vitro* was monitored by ThT assay in the presence of the reducing agent TCEP (tris(2-carboxyethyl)phosphine) (Sigma) at 20 mM and EDTA at 5 mM, plus extreme-temperature slippery PTFE Teflon beads (McMaster-Carr), over a period of 48 h in the presence or absence of recombinant FAIM (4 μM). ThT fluorescence was recorded every 10 min. Representative data from at least three experiments are shown.

The activity of FAIM in opposing mutant SOD1 aggregation in cells could be direct or indirect, the latter potentially involving other cellular elements. In order to address this issue, we established a cell free assay for evaluating FAIM function by testing the ability of FAIM to interfere with generation of mutant, ALS-associated SOD1 aggregates. We examined recombinant SOD1-G93A alone, and with FAIM, and monitored the onset of aggregation by ThT fluorescence (Yerbury et al., 2013) (excitation at 440 nm and emission at 482 nm). ThT fluorescence increases with increasing aggregation and fibril formation. We also tested native SOD1 alone and with FAIM. As shown in Figure 2, aggregates of SOD1-G93A formed over a 48 h timecourse as detected by increasing ThT fluorescence and this was largely prevented by the presence of FAIM. In contrast, native SOD1 did not aggregate and fluorescence for native SOD1 was little affected by FAIM. Thus, acting alone, FAIM is capable of interfering with the formation of mutant SOD1 aggregation.

**FIGURE 3:**
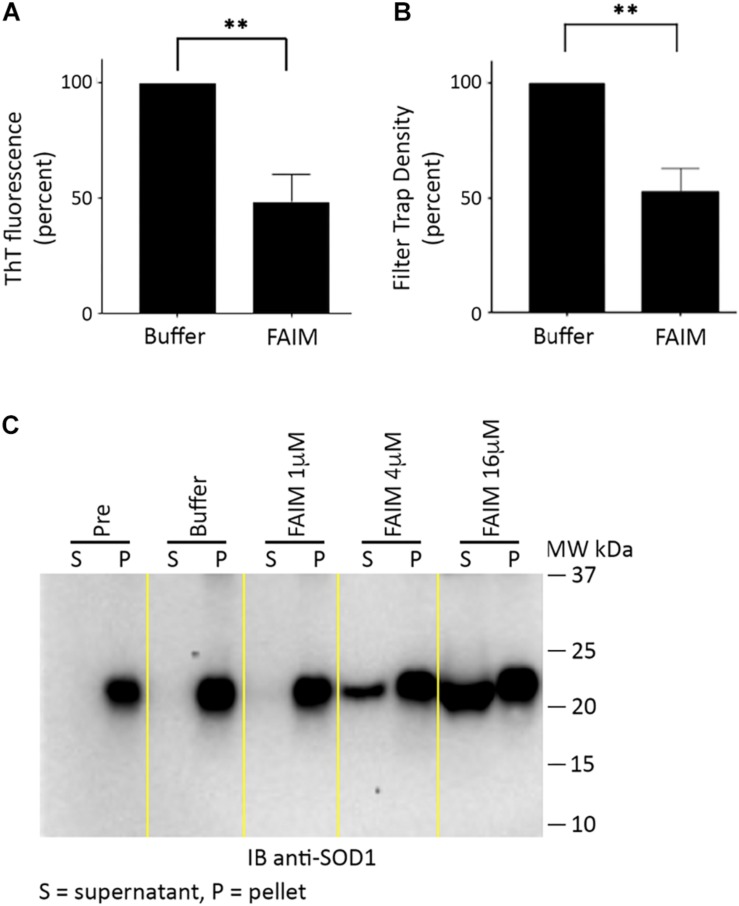
Recombinant FAIM disassembles mutant SOD1-G93A aggregates in a cell-free system. Pre-aggregated SOD1 G93A, produced as described in Methods (in the section “Generation of Pre-formed Protein Aggregates for Disaggregation Assays”), was incubated with or without 8 μM recombinant FAIM protein for 2.5 h. **(A)** Aggregation status was monitored by ThT fluorescence as described in the legend to Figure 2. Data are shown as reduction of percent ThT fluorescence compared to that of negative controls and are expressed as mean ± SEM from three independent experiments. **(B)** Aggregation status was measured by FTA, as described in the legend to Figure 1. Densitometry quantification of FTA data are shown as reduction as compared to that of negative controls and are expressed as mean ± SEM from three independent experiments. **(C)** Pre-aggregated mutant SOD1-G93A was incubated with or without recombinant FAIM protein at the micromolar doses indicated for 2.5 h, followed by centrifugation and separation of supernatant (S) and pellet (P) fractions that were subjected to SDS-PAGE under reducing conditions and immunoblotted (IB) with anti-SOD1 antibody. The locations of molecular weight markers in kDa are shown. “Pre” indicates SOD1-G93A in assembly buffer, before addition of FAIM. “Buffer” indicates Pre SOD1-G93A after addition of diluent buffer for FAIM (PBS). Digitally added vertical yellow lines were added to separate pairs of lanes representing supernatant and pellet fractions. Results shown are representative of three independent experiments.

We then examined the possibility that FAIM can act on established aggregates. To test this, we generated recombinant mutant SOD1-G93A protein aggregates in a cell free system as described in Methods (in the section “Generation of Pre-formed Protein Aggregates for Disaggregation Assays”),. We subsequently added recombinant FAIM, and monitored the level of aggregation by ThT fluorescence and by filter trap assay. No other reagents or additives (including no ATP) beyond buffer were present. We found marked reduction of protein aggregation in both assays in terms of reduced ThT fluorescence (Figure 3A) and reduced filter trapped (FTA) protein (Figure 3B), 2.5 h after addition of 8 μM FAIM, as compared to no FAIM addition (“Buffer”). These findings demonstrate the activity of FAIM in disassembling mutant SOD1 aggregates.

We further evaluated the disaggregating activity of FAIM through differential sedimentation. In this approach, SOD1-G93A protein aggregates are pelleted during high speed centrifugation (21,000 × *g*), leaving only low molecular size species or monomers in the supernatant. Mutant SOD1 in both the pellet and supernatant fractions is subsequently solubilized to monomers by SDS-PAGE prior to western blotting. As expected, pre-formed SOD1-G93A aggregates (PRE) appeared solely in the pellet fraction (P) and were solubilized in loading buffer (Figure 3C). However, we found that addition of FAIM led to a dose-dependent shift in previously aggregated mutant SOD1, which now appeared in the supernatant fraction (S), indicating dissolution to lower molecular (disaggregated) forms (Figure 3C). Addition of buffer had no effect. In other words, FAIM treatment leads to a physical shift of preformed SOD1-G93A aggregates from the sedimented pellet fraction to the non-sedimentable, soluble, supernatant fraction. In total, results from ThT, FTA and sedimentation indicate that FAIM, in the absence of any other factors, can disassemble/disaggregate protein aggregates composed of mutant SOD1.

## Discussion

FAIM was originally cloned as a molecule that inhibits Fas death receptor induced apoptosis in mouse B lymphocytes (Schneider et al., 1999). The FAIM sequence is unique, and is not related in either its short or long form of 179 or 201 amino acids, respectively (Schneider et al., 1999; Zhong et al., 2001), to two other gene products confusingly termed FAIM2 and FAIM3 by other groups (Planells-Ferrer et al., 2016). The true function of FAIM protein has been unknown for many years. In retrospect, its role was likely obscured by the lack of stress in vivarium mouse life. Recently, we showed that FAIM uniquely and non-redundantly opposes stress-induced cell death and stress-induced accumulation of protein aggregates in multiple cell types *in vitro* and in mice *in vivo* (Kaku and Rothstein, 2019). Here we show that FAIM plays a key role in cellular proteostasis, and specifically acts to prevent mutant SOD1 aggregation (in cell lines and *in vitro*) and to reverse established mutant SOD1 aggregates (*in vitro*). Thus FAIM is capable of interfering with SOD1-G93A aggregation and disassembling SOD1-G93A aggregates without the need for other cellular or soluble elements, including without the need for ATP. We hypothesize that FAIM can play a role in preventing and/or reversing dysfunctional mutant SOD1 protein aggregation that is generally acknowledged as being responsible, all or in part, for some cases of clinical FALS disease. However, other SOD1 mutations, beyond G93A, have been implicated in the pathogenesis of FALS (Saccon et al., 2013), as have mutations in other proteins, and it is important to point out that at the present time the activity of FAIM beyond SOD1-G93A has not been defined for other SOD1 mutations.

Other proteins have been shown to affect protein aggregates. Much work has been carried out with a disaggregating protein from yeast, Heat Shock Protein 104 (HSP104) by Shorter and colleagues (Torrente et al., 2016; Jackrel and Shorter, 2017), including modification to broaden and enhance its activity. But there is no vertebrate, let alone mammalian, homolog of HSP104. Although this work clearly demonstrates proof of concept, HSP104 is a foreign protein for humans and so is unlikely to represent a feasible treatment because of the expected human anti-yeast response. Further, HSP104 requires ATP for function (Schirmer et al., 1998; DeSantis et al., 2012) and it is unclear whether this would pose a functional limitation. The multiprotein combination of mammalian HSP110/70/40 (Shorter, 2011; Mogk et al., 2018) opposes protein aggregation, but the need for three different proteins is likely to limit therapeutic utility. Like HSP104, HSP110/70/40 also requires ATP for optimal activity (Shorter, 2011). Nicotinamide mononucleotide adenylyl transferase (NMNAT) in conjunction with HSP90, and the PDZ serine protease HtrA1, have been reported to disassemble some protein aggregates under limited circumstances (Jackrel and Shorter, 2017). Other proteins, including HSP27 and αB-crystallin, have been shown to interfere with protein aggregate formation, including formation of SOD1-G93A aggregates, but neither appears capable of disaggregating established aggregates (Yerbury et al., 2013). Thus, at this point in time, and to the best of our knowledge, FAIM is the only mammalian protein that works alone, without the need for ATP, to both prevent and reverse aggregation of mutant SOD1. As such there is reason to evaluate FAIM activity with respect to other aggregation-prone proteins and to determine whether FAIM has any effect on the course of ALS-like disease or other diseases in which protein aggregation is implicated in pathogenesis.

The true role of this highly conserved protein has been obscure until now because of the lack of known consensus effector/binding motifs, the lack of even partial sequence homology with any other protein, plus our finding that mice lacking FAIM evidence no obvious abnormality and experience healthy lives and normal lifespans within the confines of our specific pathogen-free animal colony (Schneider et al., 1999). Thus, it is not possible at this time to attribute the proteostatic function of FAIM to any particular structural feature. However, two studies indicated that FAIM adopts a β pleated sheet clamshell-like structure. The NMR study by Hemond et al. indicated this kind of structure is present in the C-terminal domain whereas the N-terminal domain is relatively unstructured (Hemond et al., 2009). A later X-ray crystallographic study by Li et al. (2014) indicated that both the C-terminal and N-terminal domains are arranged as clamshells. Inasmuch as SOD1 contains a β barrel (Tainer et al., 1982; Ghosh et al., 2019), these studies lead to speculation that the FAIM and SOD1 β structured regions may intercalate which might interfere with, or disrupt, aggregation. More discrete, structure-function study is likely to reveal one or more novel motifs important for opposing protein aggregation and promoting proteostasis, which may involve β structure or other structural elements.

There are a limited number of mechanisms for addressing dysfunctional and disordered proteins. These include degradation via the proteasomal system (Bett, 2016) and disposal via the autophagic pathway (Hyttinen et al., 2014), along with renaturation mediated by HSPs (Sharma et al., 2009). The failure of any of these systems to compensate for the loss of FAIM indicates that FAIM activity represents a separate, distinct, and independent pathway for dealing with proteins that are born, or made, atypical and aggregate. The very long evolutionary history of FAIM suggests the possibility that it is first among mammalian proteins that developed to counteract protein aggregation, eliminate aberrant proteins, and maintain proteostasis, and still manifests unique, non-complementary activity.

## Data Availability Statement

The datasets generated for this study are available on request to the corresponding author.

## Author Contributions

HK and TR designed the research, and analyzed and interpreted the data. HK, AL, and MG performed the research and edited the manuscript. TR wrote the manuscript.

## Conflict of Interest

The authors declare that the research was conducted in the absence of any commercial or financial relationships that could be construed as a potential conflict of interest.

## Footnotes

1 http://crispr.mit.edu
